# Ten-Year Surveillance of PCDDs/Fs and PCBs in Food and Feed from Central Italy (2016–2025): Low Contamination Levels Across Nine Food and Four Feed Categories

**DOI:** 10.3390/foods15081320

**Published:** 2026-04-10

**Authors:** Francesca D’Onofrio, Luca Alessandroni, Sesto Berretta, Laura Murru, Daniela Delfino, Fabio Busico, Alessandro Ubaldi

**Affiliations:** 1 Istituto Zooprofilattico Sperimentale del Lazio e Della Toscana M. Aleandri, Via Appia Nuova 1411, 00178 Rome, Italy; luca.alessandroni@izslt.it (L.A.); sesto.berretta@izslt.it (S.B.); laura.murru-esterno@izslt.it (L.M.); daniela.delfino@izslt.it (D.D.); fabio.busico@izslt.it (F.B.); alessandro.ubaldi@izslt.it (A.U.); 2 School of Specialisation in Food Science, Tor Vergata University of Rome, 00133 Rome, Italy

**Keywords:** PCDDs/Fs, PCBs, feed, food safety, environmental contaminants

## Abstract

This study evaluated contamination by polychlorinated dibenzodioxins and dibenzofurans (PCDDs/Fs) and polychlorinated biphenyls (PCBs) in 390 feeds and 1756 food samples collected in Latium and Tuscany (Italy, 2016–2025) using HRGC-HRMS. PCDDs/Fs and dioxin-like PCBs (dl-PCBs) are expressed as WHO 2005 toxic equivalents (WHO_05_-TEQ). Non-dioxin-like PCBs (ndl-PCBs) lack dioxin-like toxicity mechanisms due to their non-coplanar structure and are not assigned a toxic equivalence factor. Feed results were normalised to 12% moisture content. Median levels of WHO_05_-PCDDs/Fs+dl-PCBs TEQ at the upper limit in feed were 10–100 times lower than those reported in European monitoring data (EFSA, 2002–2010) for comparable categories, including additives, premixtures, raw materials and compound feed, with plant and animal feed materials below 0.03 ng/kg and aquaculture feed at 0.24 ng/kg. Food contamination was generally low, with the median WHO_05_-PCDDs/Fs+dl-PCBs TEQ 2–4 times lower than Italian national data (2013–2016), considering comparable categories such as meat, fish, milk, eggs, oils, baby foods, marine oils, animal fats and liver. Higher levels were observed in game meat, sheep products and fermented milk than in pork and poultry. The contamination remained stable over time. These results indicate an improvement in food safety thanks to national and EU regulations, although continued surveillance of high-risk and undersampled categories remains essential.

## 1. Introduction

Dioxins and PCBs pose a significant threat to food and feed safety, as demonstrated by numerous historical incidents. In Europe, industrial releases since the 1950s have caused localised contamination of food and feed, the most severe of which was the Seveso accident (1976). Later incidents, including the contamination of animal products in Germany (1997) and the major feed contamination crisis in Belgium (1999), further highlighted the risk of these contaminants entering the food chain [1,2,3,4].

In response to the Belgian dioxin crisis, the European Union adopted a risk management strategy to monitor polychlorinated dibenzodioxins (PCDDs), polychlorinated dibenzofurans (PCDFs) and polychlorinated biphenyls (PCBs) in food and feed, using a “farm-to-fork” approach [5]. The goal was to reduce the presence of these substances in the environment and the food chain, while limiting population exposure to levels below the health-based guidance value (HBGV) for dietary intake [5]. To this end, the European Commission has set an HBGV of 2 pg World Health Organisation 2005 toxic equivalents (WHO_05_-TEQ)/kg body weight per day, based on toxicological studies in animals and on reproductive and immune effects observed at doses well below genotoxic ones.

Dioxins may be introduced into the food chain through both direct and indirect pathways, originating from various environmental and technological sources [6]. Due to their lipophilic nature and resistance to metabolism, dioxins bioaccumulate preferentially in fatty tissues and biomagnify through the food chain. This sequestration mechanism underlies their toxicity, as even low-dose exposures result in lifelong retention in adipose depots [6]. Human exposure primarily occurs through the consumption of animal-based foods, particularly meat, dairy products, and fish, which together can account for up to 90% of dietary dioxin intake [6]. In livestock production, feed is the main source of exposure, with contamination originating from industrial emissions, hereditary soil and water pollution, non-industrial combustion sources (household heating, waste burning, vehicle exhausts), and, episodically, from natural events such as volcanic eruptions or fires. Due to their persistence and widespread presence, dioxins contaminate vegetation, pastures, agricultural land, and aquatic basins. In livestock production, contaminated land and grasslands used for fodder are common exposure routes, especially following flood events. Additional sources of contamination can occur during the production, processing, or transport of feed (for example, technical oils), as well as from illicit practices, such as those in the Belgian crisis. Minimising contamination during feed production and handling is essential to limit transfer into animal tissues and, ultimately, reduce human dietary exposure [7]. To manage this risk, regulatory limits for food and feed have been established: maximum levels (MLs) set by Regulation 2023/915 [8], in line with the principle “strict but achievable”, and action levels (ALs) defined by Recommendation 2014/663/EU [9] as the attention threshold that requires identification and the reduction of the source of contamination. Overall, these measures have contributed to a marked reduction in dietary exposure to dioxins and dioxin-like PCBs in Europe. For animal feed, the maximum limits (MLs) are set out in Regulation 277/2012 [10]. Monitoring of these contaminants is expensive and is ensured through periodic data provision to the competent European authorities.

This document presents a comprehensive assessment of the levels of PCDDs/Fs, dl-PCBs, and ndl-PCBs, as well as the distribution of their congeners, in food, feed, and feed premixes from the Lazio and Tuscany regions (Italy), based on a 10-year dataset (2016–2025). The main purpose of the study is to provide an updated dataset covering a recent time period than existing EFSA monitoring data [11], while including various types of matrices (nine food matrices and four feed matrices) within the same study, representing a broader approach than other studies reported in recent scientific literature [7,12,13,14,15,16,17,18,19,20]. The study assesses differences in contamination levels across different product categories, with a particular focus on animal-derived and dairy products. Finally, the results are compared with those reported in the scientific literature [7,11,12,13,14,15,16,17,18,19,20], in order to identify possible temporal or regional trends, thereby contributing to the improvement of risk management strategies for food and feed safety.

## 2. Materials and Methods

### 2.1. Study Area and Samples

This study presents data on food and feed samples collected during official control activities carried out in the Latium and Tuscany regions between 2016 and 2025. The samples were analysed by the Istituto Zooprofilattico Sperimentale del Lazio e della Toscana M. Aleandri (IZSLT) and were convenience samples, not selected according to a statistically designed monitoring plan, collected from various sources, including retail outlets, farms, and import entry points.

A total of 1756 food samples were analysed for the presence of polychlorinated dibenzo-p-dioxins, polychlorinated dibenzofurans, dioxin-like (dl) and non-dioxin-like (ndl) polychlorinated biphenyls (PCBs). Food samples were classified into the following categories: meat and meat products; fishery products and bivalve molluscs; marine oils (mainly omega-3 supplements); raw milk and dairy products; eggs; vegetable oils; animal fats; baby foods for infants and young children; and liver.

In addition to food samples, 390 feed samples were analysed and classified as additives, premixes, raw materials, and compound feed. All data were analysed as a single dataset, regardless of the origin of the monitoring plan, to assess overall contamination levels across food and feed categories. The sampling frequencies for each of the analysed matrices are reported in Table 1.

### 2.2. Reagents and Standards

All reagents and solvents used in the analysis were tested and confirmed to be free of contamination at levels of interest. The columns in the Power-Prep automated system, along with multilayer silica, alumina, and carbon, were supplied by Lab Service Analytica (Bologna, Italy). Dichloromethane for trace analysis, n-hexane 99% RPE, toluene RPE analytical grade, and acetone RPE analytical grade were obtained from Carlo Erba Reagents s.r.l (Cornaredo, MI, Italy).

All standard ^13^C-labelled recovery, clean-up, and injection standard solutions were provided by CIL (Cambridge Isotope Laboratories, Tewksbury, MA, USA). The EPA 1613 PCDDs and PCDFs calibration solutions (CS1–CS5) were also supplied by CIL (Cambridge Isotope Laboratories, Tewksbury, MA, USA). Calibration for dl-PCBs was performed in-house using a mixture supplied by CIL (Cambridge Isotope Laboratories, Tewksbury, MA, USA) and two ^13^C-labelled mixtures (surrogate and recovery standards) supplied by Wellington Laboratories.

### 2.3. Analytical Method

The analyses were carried out using a validated and accredited method (EN ISO/IEC 17025 [21]), routinely employed for determining PCDDs/Fs and PCBs in food and feed. This method complies with international standards and Regulation (EU) 2017/644 [22], and has been successfully evaluated in proficiency testing. Measurements were performed by High-Resolution Gas chromatography coupled to High-Resolution Mass Spectrometry (HRGC-HRMS), using isotope-labelled internal standards.

A total of 35 analytes were determined: 10 PCDDs, 7 PCDFs, 12 dioxin-like PCBs (8 mono-ortho and 4 non-ortho), and 6 non-dioxin-like PCBs listed in Table 2.

The method fully complies with EU legislation [22] (Regulation 2017/644) and aligns with US EPA 1613 procedures [23] for the purification of extracts.

For sample preparation, ^13^C-labelled standards (surrogate standard) were added to the homogenised samples, which were then lyophilised and extracted using an automatic Soxhlet apparatus, usually with a hexane-acetone mixture. After the extract was evaporated, the residue was weighed to determine the fat content. Analytical procedures were adapted to the different matrices: for fishery products, bivalve molluscs, liver, baby food, and feed, results are expressed on a wet-weight basis without gravimetric lipid determination, as required by current regulations [8]. For feed, the moisture content was determined gravimetrically by weighing two separate aliquots (approximately 5.000 g each, weighed to the nearest milligram) before and after drying in an oven at 103 °C. Results were normalised to 12% moisture, in accordance with EU legislation, to allow comparison with MLs [10].

The extract was purified in two steps: first, the lipid matrix was carbonised on a silica gel acidified with sulfuric acid; then, automated chromatographic separation (PowerPrep^®^, LabService Analytica Srl, Anzola dell’Emilia, Italy) was performed on acid-basic silica, alumina, and carbon columns. This process yielded two distinct extracts: one containing PCDDs/Fs and non-ortho-PCBs (nPCBs), and the other containing the remaining PCBs. The extracts were evaporated in two steps using dodecane as a keeper. The final volumes, after adding the syringe standards, were 20 μL for PCDDs/Fs and non-ortho-PCBs and 80 μL for PCBs.

### 2.4. Instrumental Analysis

Instrumental analysis was performed using a High-Resolution Gas chromatographic system coupled to a high-resolution DFS (Thermo Fisher Scientific, Waltham, MA, USA) magnetic-sector mass spectrometer with a mass resolution of R ≥ 10,000. Chromatographic separation of PCDDs and PCDFs was performed on a 60 m × 0.25 mm × 0.25 μm film thickness ZB-semivolatile capillary column (Phenomenex, Torrance, CA, USA). For dl-PCBs, a 60 m × 0.25 mm × 0.25 μm film thickness XLB capillary column (Phenomenex, Torrance, CA, USA) was used. Congener identification was based on exact masses, isotope ratios, and retention times relative to the labelled standards. Quantification was achieved using five-point calibration curves in accordance with US EPA 1613 [23]. The concentration ranges of the calibration curves were 0.03–1 pg/μL for PCDDs/Fs, 0.3–10 pg/μL for n-PCBs, and 0.5–50 pg/μL for ndl and dl-PCBs. The method was validated in accordance with Regulation (EU) 2017/644 [22]: linearity, specificity, limits of detection and quantification (LODs and LOQs), repeatability, reproducibility, and trueness were found to comply with the expected limits. The laboratory regularly ensures the accuracy of its results by participating in proficiency tests organised by the European Reference Laboratory for Halogenated Persistent Organic Pollutants in Food and Feed every six months.

### 2.5. Data Analysis and Statistics

In accordance with European legislation, analytical results for dioxins, dl-PCBs and their sum were reported as values “upper limit”, meaning that undetected concentrations were set equal to the LOQ. The toxic equivalence factors (TEFs) proposed by WHO in 2005 [24] were used to calculate WHO toxic equivalents (WHO_05_-TEQ). Although WHO is currently developing updated TEF values [25], the 2005 TEFs remain the regulatory standard for food safety assessment in the European Union (EU) and were therefore applied in this study. To facilitate comparison with regulatory limits, the following sums were calculated: PCDDs/Fs, dl-PCBs, and PCDDs/Fs+dl-PCBs, all expressed as upper limits. Results were reported in WHO_05_-TEQ: picograms per gram of fat weight (pg WHO_05_-TEQ/g fw) for most matrices. For fishery products, bivalve molluscs, liver, and baby food, results were expressed per gram wet weight (pg WHO_05_-TEQ/g ww). For feed, results were reported in ng WHO_05_-TEQ/kg feed, normalised to 12% moisture content, in accordance with EU legislation [10].

For non-dioxin-like PCBs (ndl-PCBs), concentrations were reported in ng/g lipid for most matrices, in pg/g wet weight for fishery products, bivalve molluscs, liver, and baby food, and in μg/kg feed (normalised to 12% moisture) for feed, in line with EU requirements [8]. To study congener patterns, the levels of congeners PCDDs, PCDFs, and dl PCBs were analysed without transformation into TEQ [26]. To facilitate comparison with data from other countries, samples of animal origin with a fat content below 2% were excluded from the analysis. According to EU legislation, samples with a fat content below 2% must be reported on a wet-weight basis rather than on a fat basis. Because this prevents direct comparison with samples expressed on a fat basis within the same food category, these samples were excluded from the analysis. In such cases, the maximum levels expressed on a fat basis are converted using a factor of 0.02.

For this study, in order to compare the profiles of the most contaminated samples, “compliant” was defined as any sample below the action limit (AL) or ML, and “non-compliant” referred to exceeding these thresholds, taking into account the uncertainty in assessing legal compliance. According to Regulation 2017/644 [22], the extended measurement uncertainty (U) is used and calculated using a coverage factor of 2. The sum of the estimated extended uncertainties of the separate analytical results of PCDDs/Fs and/or PCBs is used to calculate the extended uncertainty of the sum.

Statistical analyses were performed using Jamovi (version 2.6, The Jamovi Project, Sydney, Australia) [27], built on the R statistical environment (version 4.4, R Core Team, Vienna, Austria) [28]. Data visualisation and graphical representations were generated using Microsoft Excel.

Feed samples were divided into four main categories: additives, premixtures, raw materials and compound feed. For each category, the mean, median, and percentiles (25th and 75th) of the sums of PCDDs/Fs, PCBs (dl- and ndl-) and PCDDs/Fs+dl-PCBs were calculated. Since additives and premixtures had few samples and generally low values, statistical comparisons using the Kruskal–Wallis test were applied only to raw materials and compound feed. Raw materials were further classified by origin (vegetable, animal and mineral), while compound feed was divided into complete and complementary. The highest contamination values were observed in animal raw materials and complete feeds intended for aquaculture. For these two matrices, which exhibited the highest levels of contamination, the distribution of individual PCDDs/Fs and PCB congeners (dl- and ndl-) was also analysed.

Regarding the food samples, the study considers nine categories. For each category, the calculated means, medians, and percentiles (25th and 75th) of the sums of PCDDs/Fs, PCBs (dl- and ndl-), and PCDDs/Fs+dl-PCBs were analysed. In categories where the number of samples permitted (meat, milk and derivatives, and fish and molluscs), comparisons were made at the species level. Significant differences were observed across the three categories; therefore, pairwise comparisons were conducted using the Dwass–Steel–Critchlow–Fligner (DSCF) test, a non-parametric post hoc procedure that is robust to unequal variances and sample sizes.

### 2.6. Estimation of Adult Intake of PCDDs/Fs and dl-PCBs

The measured concentrations of contaminants in food samples were combined with data on average daily food consumption derived from two Italian National Food Consumption Surveys conducted by the Italian National Institute of Food and Nutrition Research [29] and by the Council for Agricultural Research and Economics (CREA) [30]. An average adult body weight of 70 kg was assumed, consistent with the approach used by the European Food Safety Authority [31]. The INRAN dataset was selected to ensure comparability with previous national exposure assessments [13,14]. The estimated daily intake (pg WHO_05_-TEQ/kg bw/day) was calculated by multiplying the mean contaminant concentration in each food category by the corresponding mean daily consumption, then dividing by body weight. Fat-content values were derived from the Italian food composition database CREA [32] and assigned at the species and product level. For each species or product subcategory, the mean contaminant concentration was converted to a wet-weight basis using the corresponding species-specific fat-content value. Where the dietary surveys reported consumption data disaggregated by species or product type, the wet-weight concentration for each subcategory was multiplied by the corresponding mean daily consumption; the resulting intake values were then averaged to obtain a single estimate per food category. For meat and meat products, fresh meat and processed products (salumi) were treated separately, using distinct fat-content values reflecting their different lipid compositions. For milk and dairy products, fat content values were assigned separately by species.

## 3. Results

### 3.1. Feed Results

**Figure 1:**
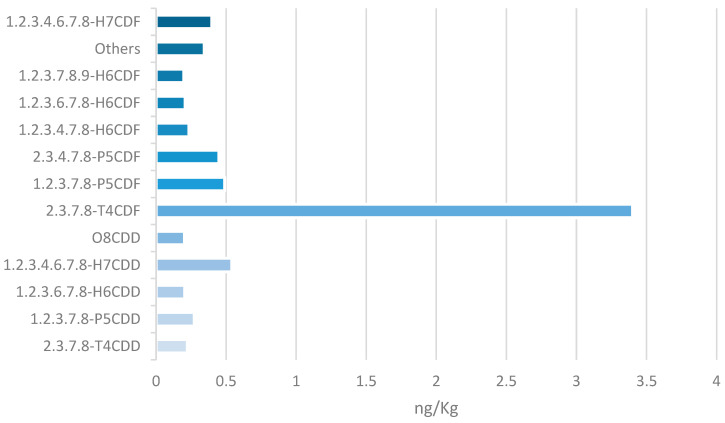
PCDDs/Fs pattern in the raw material non-compliant sample. In “others” are the included congeners at ≤2%: 1.2.3.4.7.8-H6CDD; 2.3.4.6.7.8-H6CDF; 1.2.3.4.7.8.9-H7CDF (1%); and 1.2.3.7.8.9-H6CDD and O8CDF (2%).

In total, 390 feed samples were analysed during the study period (2016–2025). Among these, only one plant-based feed material (barley silage), analysed in 2017, was found to be non-compliant with the dioxin limits (WHO_05_-PCDDs/Fs-TEQ), even after subtracting measurement uncertainty (corrected value: 0.84 ng WHO_05_-TEQ/kg). In this sample, the sum of PCDDs/Fs and dl-PCBs was below the regulatory limit of 1.25 ng WHO_05_-TEQ/kg [10] when measurement uncertainty was taken into account (1.16 ng WHO_05_-TEQ/kg). Overall, only one non-compliant sample was found, corresponding to 0.6% of the raw materials (1/165) and 0.26% of the total 390 feed samples analysed (1/390), thus indicating a low prevalence of regulatory exceedances. The PCDDs/Fs congener profile (Figure 1) was determined for the single non-compliant plant-based feed sample to characterise the relative contribution of individual PCDDs/Fs congeners to the exceedance. The PCDDs/Fs profile was dominated by furans, particularly 2,3,7,8-TCDF, while OCDD levels were comparatively low. The levels of congeners (PCDDs, PCDFs, and dl PCBs) were analysed without transformation into TEQ [7,26].

Table 3 summarises the statistical descriptors of PCDDs/Fs, dl-PCBs and 6 ndl-PCBs across the different feed categories analysed. For each contaminant group, the median, 25° percentile (P25), and 75° percentile (P75) are reported. For feed, results are reported in ng WHO_05_-TEQ/kg feed, normalised to a moisture content of 12%, in accordance with EU legislation [10].

**Figure 2:**
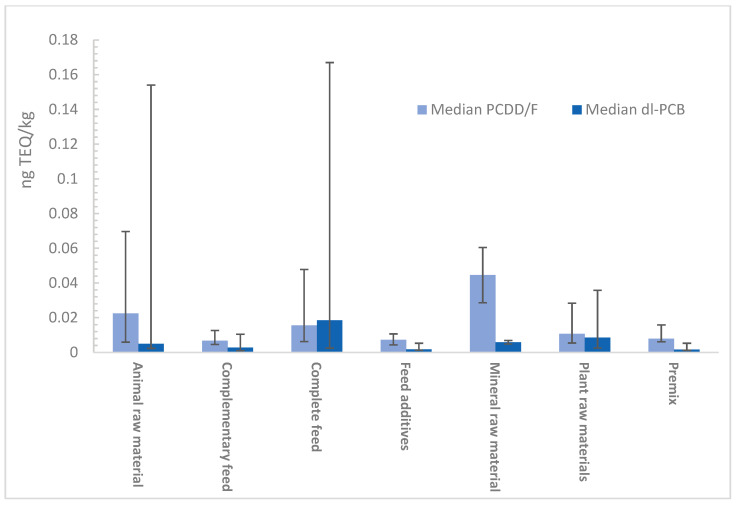
Contribution of PCDD/Fs and dioxin-like PCBs to total WHO-TEQ in different feedstuff categories. Bars represent median values; error bars indicate interquartile ranges (IQR). Sample sizes: animal raw materials (n = 29), plant raw materials (n = 135), mineral raw materials (n = 2), complete feed (n = 129), complementary feed (n = 74), feed additives (n = 11), premix (n = 10). Categories with a limited sample size (n < 5) should be interpreted with caution.

Contaminant levels in different feed categories were generally low. The highest concentrations appeared in raw materials and compound feeds, while additives and premixtures had lower levels; however, the limited sample sizes for these categories (n = 11 and n = 10) hindered robust statistical comparisons. Greater variability was observed in raw materials and compound feeds, which were further analysed by subcategory using the Kruskal–Wallis and Dwass–Steel–Critchlow–Fligner (DSCF) post hoc tests (Tables in Appendix A Table A1 and Table A2). In raw materials, no significant differences were identified between plant, animal, and mineral origins, despite slightly higher values in animal-derived materials, influenced by some high observations. In compound feeds, the complete feeds exhibited significantly higher contamination than the complementary feeds. Among the subcategories, aquaculture feed showed the highest levels, whereas cattle feed demonstrated the lowest. Figure 2 summarises the contribution of PCDD/Fs and dioxin-like PCBs to the total WHO_05_-TEQ. Animal feedstocks showed higher median PCDD/F levels with substantial variability, while dl-PCBs remained low across all categories. Complete feeds consistently showed higher contamination than complementary feeds for both classes of contaminants. Non-dioxin-like PCBs were detected at low levels in all categories with minimal changes and are therefore not shown.

Congener-specific analysis of aquaculture feed and animal feedstocks found that most dioxins and furans remained close to or below the LOQ. However, LOQs were determined individually for each sample and congener using the instrumental software. The LOQ is calculated as: LOQ = ((SUMN × hdlf × SPAis)/(SUMHIS × ARFA)) × hDF, where SUMN is the sum of noise, hdlf is the detection limit, SPAis is the internal standard amount, SUMHIS is the sum of internal standard heights, ARFA is the actual response factor, and hDF is the dilution factor. Both matrices showed sporadic contamination peaks, particularly in highly chlorinated congeners (O8CDD, O8CDF) and dl-PCBs (PCB 126: maximum 40.5 pg/g in raw materials; maximum 6.8 pg/g in aquaculture feed). Despite high variability among samples, the overall toxicological risk remains low, though the peaks underscore the need for continuous monitoring.

### 3.2. Food Results

**Figure 3:**
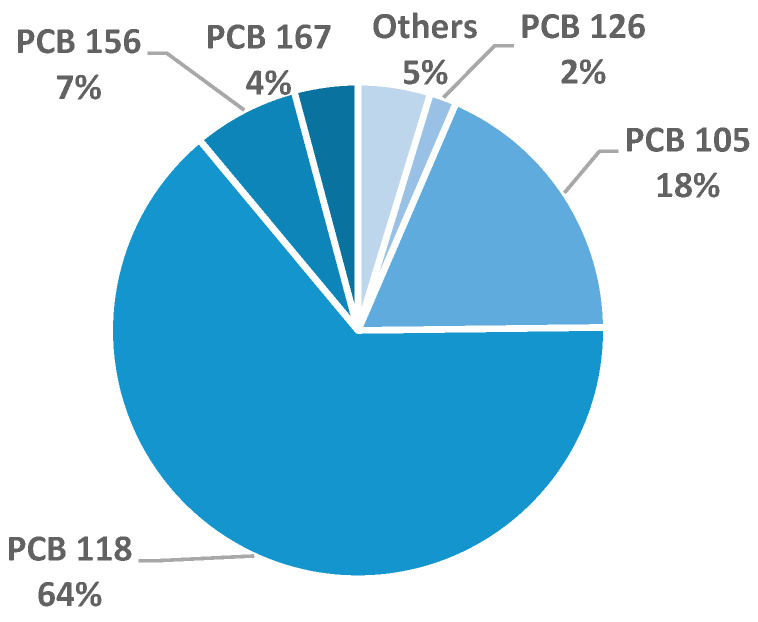
Congener profile of dl-PCBs in the non-compliant yoghurt sample. “Others” included congeners at ≤1%: PCB 77, PCB114, PCB157, PCB 189 (1%); PCB 81, PCB 169, PCB 123 (<1%).

Among 1756 food samples, eight (0.46%) exceeded regulatory limits. Most non-compliance was due to PCBs: six samples exceeded the action levels for dl-PCBs (two milk and dairy products, two meats, two fish/molluscs). A yoghurt sample collected in 2016 had a sum of PCDD/Fs and dl-PCBs of 4.6 pg WHO_05_-TEQ/g fw. At the time of sampling, it complied with the previous limit of 6 pg WHO_05_-TEQ/g fw (Reg. 1881/2006) [33], but now exceeds the current limit of 4 pg WHO_05_-TEQ/g fw (Reg. EU 915/2023) [8]. A vegetable oil sample collected in 2020 exceeded both the intervention level and the current legal upper limit for dioxins and furans (0.89 pg WHO_05_-TEQ/g fw versus 0.75 pg WHO_05_-TEQ/g fw) as well as the sum of PCDD/Fs and dl-PCBs (1.4 versus 1.25 pg WHO_05_-TEQ/g fw). Two milk samples with ndl-PCBs above 40 ng/g fw were below the limit after applying measurement uncertainty (37 and 39 ng/g fw), and one liquid-adapted milk sample exactly met the current ndl-PCBs limit of 1 ng/g wet weight, thus remaining formally compliant. In the non-compliant yoghurt (2016), the exceedance of the regulatory limit was driven by dl-PCB congeners, as shown in Figure 3, highlighting the congeners that contributed most to contamination. The congener profile of the non-compliant yoghurt sample was determined to illustrate the relative contributions of dl-PCBs. PCB 118 was the dominant dl-PCB, followed by PCB 105, PCB 156, and PCB 167, which together accounted for over 90% of total dl-PCBs, consistent with reported congener patterns in the milk of dairy cows experimentally exposed to contaminated feed [34].

Table 4 summarises the statistical descriptors for PCDD/Fs, dl-PCBs and 6 ndl-PCBs across the analysed food categories. For each contaminant group, the median, 25th percentile (P25) and 75th percentile (P75) are reported. Values are expressed as pg WHO_05_-TEQ/g fw for lipid-based matrices, or per g wet weight (ww) for fish, bivalve molluscs, liver and baby food, in accordance with EU legislation [8].

Organochlorine contaminant levels varied considerably across food categories, with the highest total WHO_05_-TEQ concentrations in dairy products and marine oils, followed by eggs and meat. Baby foods showed very low values, consistent with more stringent production practices and quality controls throughout the production chain for food intended for infants and young children.

**Figure 4:**
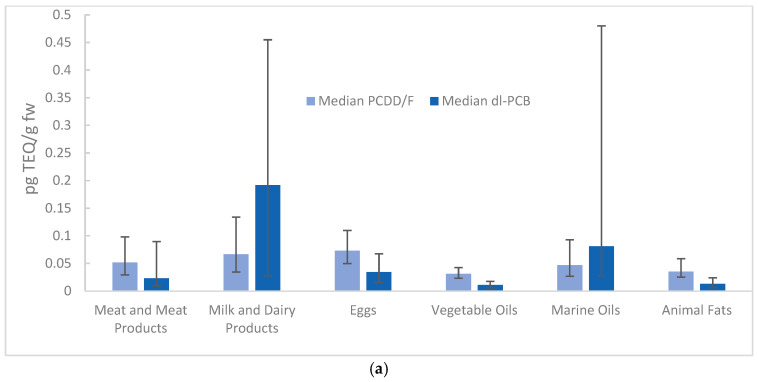

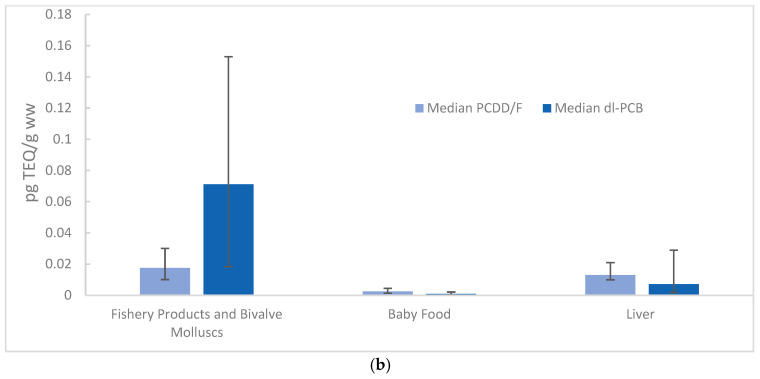
Median WHO_05_-TEQ concentrations by food category with interquartile range (IQR) error bars (median–P25; P75–median). Results are presented on a fat basis for fatty matrices (pg TEQ/g fw; panel (**a**)) and on a wet weight basis for matrices regulated accordingly (pg TEQ/g ww; panel (**b**)).

**Figure 5:**
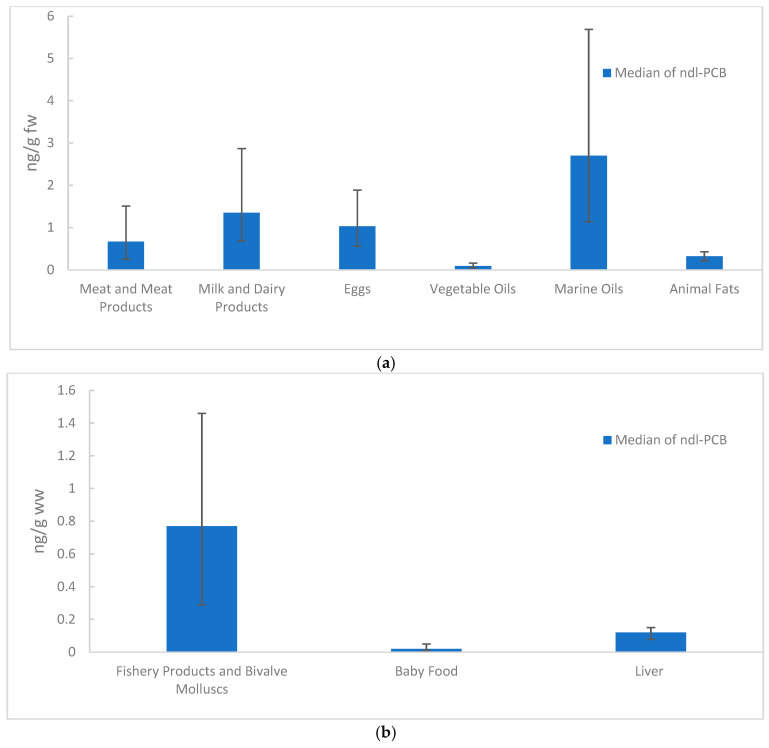
Median 6-ndl-PCB levels by food category with IQR error bars (median–P25; P75–median). Fatty matrices are shown on a fat basis (pg TEQ/g fw; panel (**a**)), while matrices regulated on a wet weight basis are shown on a wet weight basis (pg TEQ/g ww; panel (**b**)).

The relative contribution of contaminant classes varied by food type. PCDDs/Fs were most abundant in eggs and dairy products, whereas dl-PCBs accumulated mainly in dairy products. Non-dioxin-like PCBs were highest in marine oils and dairy products and showed considerable variability, particularly in marine oils and eggs. Plant-based foods, such as vegetable oils, consistently showed lower contamination across all contaminant classes, reflecting their preferential bioaccumulation in animal products. Figure 4 and Figure 5 illustrate contamination patterns using the median and interquartile range. This approach minimises the influence of extreme values and provides a clearer visual comparison across food categories. WHO_05_-TEQ values (PCDDs/Fs and dl-PCBs combined) and ndl-PCBs are presented separately due to their different concentration scales. TEQ results are expressed on a fat basis (pg TEQ/g fw) for fatty matrices (e.g., meat, dairy) and on a wet weight basis (pg TEQ/g ww) for matrices regulated accordingly (e.g., fish, baby food).

Temporal trends were assessed for six food categories with sufficient annual coverage (meat, dairy products, eggs, baby foods, vegetable oils, and fish). Three categories were excluded: liver and animal fats due to insufficient or highly irregular temporal coverage (liver: n = 16 in 5 non-consecutive years; animal fats: n = 53 with annual sample sizes ranging from 0 to 16), and marine oils due to low and variable annual sample sizes (n = 1–6/year).

The median WHO_05_-TEQ remained relatively stable from 2016 to 2025 across the six categories. Fluctuations were observed from year to year, but are likely attributable to differences in sample origin, matrix heterogeneity, and compositional diversity within categories rather than actual temporal trends, given the convenience sampling design and variable annual sample sizes. No formal statistical analysis of trends was performed. Although marine oils (omega-3 supplements) were excluded from this assessment, recent samples showed higher contamination levels (2024: median 0.528 pg WHO_05_-TEQ/g fw, n = 5; 2025: median 0.885, n = 4) compared to previous years (2016–2022: range 0.056–0.25 pg WHO_05_-TEQ/g fw, n = 1–6/year). Although these observations were based on small sample sizes and required cautious interpretation, these findings suggested that marine oil supplements could be subjected to targeted surveillance as potential contributors to dietary exposure. The apparent increase is likely driven by batch- and brand-related variability under limited, non-uniform sampling.

**Figure 6:**
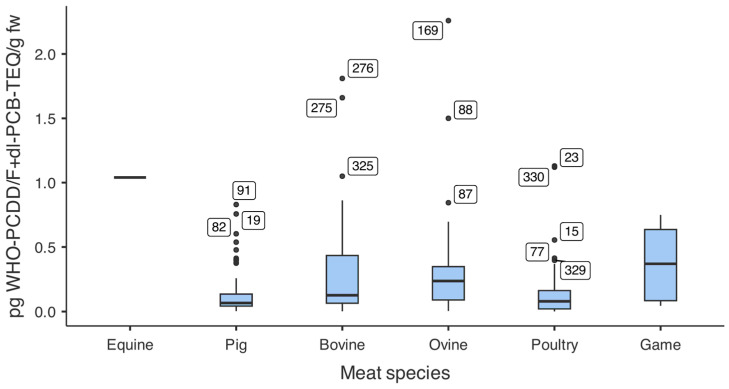
WHO_05_-PCDDs/Fs+PCBs-TEQ levels in different meat types. Boxes represent interquartile ranges (25th–75th percentiles); horizontal lines within boxes indicate medians; whiskers extend to 1.5 × IQR; individual points represent outliers. Sample sizes: horse (n = 1), pork (n = 146), beef (n = 82), sheep (n = 51), poultry (n = 107), game (n = 9).

Species-level analysis was conducted for milk, meat, and fish, which had sufficient sample sizes and substantial variability in contaminant levels. Other categories (e.g., liver, oils) were reported only at the macrocategory level due to limited sample sizes or low contamination levels. Figure 6 shows WHO_05_-PCDDs/Fs+dl-PCBs-TEQ distributions across meat types. Game meat had the highest median contamination, followed by ovine and bovine, while pork and poultry had the lowest levels. Pairwise comparisons indicated similar levels between ovine and bovine, both significantly higher than pork and poultry, which did not differ from each other. Results for game and horse meat were not included in statistical comparisons due to limited sample sizes.

A small proportion of high-value outliers (2.3% of meat samples) was identified across categories without clear temporal or geographic clustering, suggesting isolated contamination events. Detailed comparisons are provided in Appendix A Table A3.

**Figure 7:**
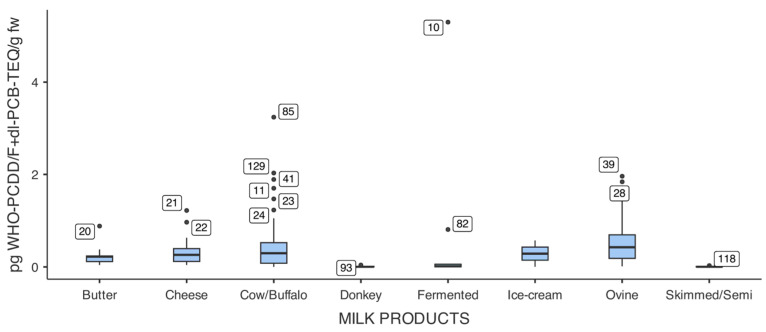
WHO_05_-PCDDs/Fs+dl-PCBs-TEQ levels in milk and dairy products. Boxes represent interquartile ranges (25th–75th percentiles); horizontal lines indicate medians; whiskers extend to 1.5 × IQR; points show outliers. Sample sizes: fermented milk (n = 9), ovine milk (n = 82), cow/buffalo milk (n = 79), cheese (n = 27), butter (n = 13), donkey milk (n = 4).

Figure 7 shows the distribution of WHO_05_-PCDDs/Fs+dl-PCBs-TEQ levels (pg/g fw) across various milk and dairy product categories. Overall contamination was low, with median values generally below 0.5 pg WHO_05_-TEQ/g fw in all categories. As shown in Figure 7, 14 samples were identified as outliers because their WHO_05_-PCDDs/Fs+dl-PCBs-TEQ values exceeded the overall distribution. Most outliers occurred in 2016 (71%, 10/14); some were imported products, while others were farmed samples collected from the Latium and Tuscany regions. Species-level analysis, excluding categories with fewer than 10 samples, revealed significant differences in contamination levels among dairy products (Kruskal–Wallis test, *p* = 0.01). Although the Kruskal–Wallis test revealed overall significant differences among product categories (*p* = 0.01), the DSCF post hoc test did not identify statistically significant pairwise differences. Notably, comparisons involving ovine products consistently approached but did not reach significance (*p* ranging from 0.068 to 0.094), suggesting a trend that may warrant further investigation with larger sample sizes, particularly for butter (n = 13) and cheese (n = 27).

**Figure 8:**
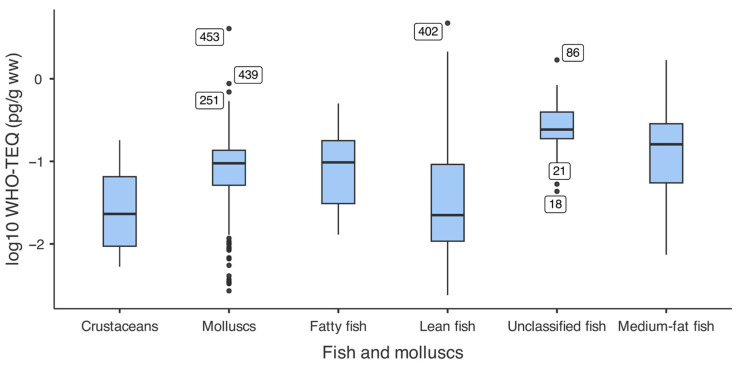
WHO_05_-PCDDs/Fs+dl-PCBs-TEQ levels in fish and molluscs (log_10_ scale). Boxes show interquartile ranges with median lines; points indicate outliers. Logarithmic scale used for visualisation; analyses performed on untransformed data. Categories: crustaceans (n = 16), molluscs (n = 299), fatty fish (e.g., salmon, eel; n = 14), lean fish (e.g., cod, hake; n = 65), unclassified (heterogeneous 2016–2017 samples; n = 25), medium-fat fish (e.g., sea bass, trout; n = 85).

Significant differences in contamination levels were observed across fish and mollusc categories (Kruskal–Wallis W = 80.8, *p* < 0.001). Due to the wide range of values and skewed distributions, WHO_05_ PCDDs/Fs+dl-PCBs-TEQ data are shown on a logarithmic scale in Figure 8, while statistical analyses were performed on untransformed data. Five higher outliers were identified; these values originated from a combination of imported and local samples, consistent with isolated contamination events rather than systemic contamination.

Unclassified fish (primarily from the 2016–2017 sampling, n = 25) had the highest median WHO_05_-TEQ (0.24 pg/g ww), significantly higher than lean fish (*p* < 0.001), molluscs (*p* < 0.001), and crustaceans (*p* < 0.001). These samples originated from multiple local aquaculture sites across Latium and Tuscany rather than a single geographic location, indicating that the observed high values reflect variability within the category rather than a localised contamination event. The elevated levels may be related to feed quality or management practices at individual aquaculture sites, though the available data do not allow further attribution. Medium-fat fish showed intermediate contamination (0.161 pg/g ww), significantly higher than lean fish (*p* < 0.001) and crustaceans (*p* < 0.001). Molluscs (0.095 pg/g ww) were significantly more contaminated than lean fish (*p* < 0.001) and crustaceans (*p* = 0.004), representing the second-highest category.

Unexpectedly, fatty fish showed a low median contamination (0.098 pg/g ww) that was not significantly different from lean fish (*p* = 0.225), likely due to the prevalence of farmed salmon in the sample set (n = 11/14), which benefited from improved aquaculture feed quality. Lean fish and crustaceans displayed the lowest and most similar contamination levels (*p* = 0.999). Detailed pairwise comparisons are provided in Appendix A Table A5.

### 3.3. Estimated Adult Intake of PCDDs/Fs and dl-PCBs

Estimated daily intakes of dioxins and dl-PCBs for adults were calculated by combining mean contaminant concentrations measured between 2016 and 2025 with food consumption data from two Italian surveys (INRAN 2005–06 [29] and CREA 2018–2020 [30]). Only absolute intake values (pg WHO_05_-TEQ/kg bw/day) are presented; differences between surveys reflect variation in dietary patterns rather than temporal changes in contamination. Fish and seafood were the largest contributors to total intake in both surveys, followed by cheese and milk. Butter, vegetable fats, and eggs contributed less. To provide further context, the relative contribution of each food category to total intake was calculated (Table 5).

These descriptive estimates allow for an assessment of the relative importance of different food categories in determining exposure.

## 4. Discussion

### 4.1. Feed

Comparison with the recent literature data on feed contamination indicates that PCDDs/Fs and dl-PCBs levels detected in this study were substantially lower than those previously reported. Median WHO_05_-TEQ levels in raw materials were one to two orders of magnitude lower than the values reported by Pajurek et al. [12]. Specifically, plant materials showed median values of 0.011 ng PCDDs/Fs-TEQ/kg and 0.0085 ng dl-PCBs-TEQ/kg (vs. 0.26 and 0.030 ng/kg in Pajurek, 2024), while animal raw materials exhibited median values of 0.022 ng PCDDs/F-TEQ/kg and 0.0049 ng dl-PCB-TEQ/kg (vs. 0.32 and 0.68 ng/kg for animal fats in Pajurek, 2024). Similarly, ndl-PCBs concentrations in animal materials were approximately 60-fold lower (median 0.10 vs. 5.85 μg/kg).

Animal raw materials showed a highly skewed distribution, with very low medians (0.024 ng WHO_05_-PCDDs/Fs+dlPCBs-TEQ/kg) but occasional values that raised the mean (0.069 ng WHO_05_-PCDDs/Fs+dlPCBs-TEQ/kg), indicating that contamination is specific rather than systemic, affecting only a limited number of batches.

Similarly, in compound feeds, contamination levels were markedly lower than those reported in the literature. Complete feeds for aquaculture had a median WHO_05_-PCDDs/Fs+dlPCBs-TEQ/kg of 0.24 ng/kg (mean 0.32 ng/kg), approximately 2.6-fold lower than the 0.63 ng WHO_05_-PCDDs/Fs+dlPCBs-TEQ/kg reported by Pajurek et al. [12] for pet and fish feeds. An even more pronounced reduction was observed in terrestrial animal feeds (median: 0.017 ng WHO_05_-PCDDs/Fs+dlPCBs-TEQ/kg). Dl-PCBs were the primary contributor to total WHO_05_-TEQ in aquaculture feeds (median 0.20 vs. 0.055 ng/kg for PCDDs/Fs), consistent with the use of fish-derived ingredients, although absolute levels remained very low. Concentrations of ndl-PCBs were likewise reduced (median 1.86 vs. 5.02 μg/kg in Pajurek, 2024).

These differences may reflect the different sampling periods (2016–2025 in our study versus 2013–2018 and 2022 in the Polish study) and the geographic origin of the samples. In the Polish study, samples were collected across production plants, distributors, and farms throughout Poland as part of the National Official Feed Control Plan.

The TCDF (2,3,7,8-tetrachlorodibenzofuran) dominated the congener profile observed in non-compliant silage differs significantly from typical background environmental contamination, where OCDD usually predominates. This pattern is consistent with contamination from heat treatment or recent combustion sources, as previously reported by Pajurek et al. [7] for feed materials subjected to drying or heat treatment.

Overall, these findings demonstrate a marked reduction in feed contamination compared to earlier surveillance data (2002–2010) [11]. This likely reflects the combined effects of stricter regulatory limits, improved ingredient sourcing, and reduced environmental contamination.

### 4.2. Food

The EFSA scientific report on dioxin, furan and PCB levels in food and feed [11], covering the period 2002–2010, reported higher mean concentrations than those observed in our samples (2016–2025). This decreasing trend in our samples likely reflects the effects of stricter regulations and more effective controls on contamination by these compounds. In our most recent samples (2016–2025), total levels of dioxins, furans and dioxin-like PCBs (WHO_05_-TEQ) in foods were generally lower than those reported by Diletti et al. for the period 2013–2016, which included samples from the entire Italian country [13]. Eggs, milk and meat show median levels approximately 2–4 times lower, with a significant reduction in both PCDD/Fs and dl-PCBs. Even in fish, although dl-PCBs still represent the main contribution to TEQ, total values are approximately 3–4 times lower than previous data [13]. This reduction is likely related to improved aquaculture feed quality, including the use of less contaminated fish meal and oils, as well as partial replacement with plant proteins and vegetable oils. In land animal livers, total levels were particularly low in our dataset, likely because it includes several species, while Diletti et al. [13] considered only sheep liver samples, which are known to accumulate higher levels of contaminants. Vegetable oils showed minimal variations, suggesting stable levels over time. The patterns observed across different food categories and species are consistent with previous reports. Among meat, the highest values are observed in sheep and game, while poultry and pigs have lower concentrations, a parallel that confirms Diletti’s results for the Italian data. Similarly, in milk and dairy products, the highest values were associated with sheep milk, while butter showed lower values, following the same accumulation pattern previously reported [13]. Overall, these results indicate a decreasing trend in the exposure of the Italian population to PCDD/Fs and dl-PCBs in food, although our data are limited to two regions, while Diletti’s study represents the national situation. At the regional level, compared to Barone et al. [14,15,20], the differences are more likely attributable to geographical origin, since those studies focused on southern Italy, a region characterised by a higher industrial load and historically higher background environmental contamination than central Italy. Comparability with our dataset is also limited by the narrow sampling windows of such studies (two to three months each) compared to our 10-year dataset, which is less susceptible to batch- and season-related variability. In contrast, Giannico et al. [18] reported median WHO_05_-PCDDs/Fs+dl-PCBs-TEQ of 0.83 pg/g fat in raw milk and 0.62 pg/g fat in dairy products from farms located within 20 km of the Taranto industrial area (2013–2018), values higher than our overall median of 0.32 pg/g fat but significantly lower than those reported by Barone et al. [14] for the same region. It should be noted that the Giannico study was specifically designed as a targeted surveillance programme around a major industrial site, which may explain the higher levels observed compared with our samples. The median ndl-PCBs were 1.92 ng/g fat in raw milk and 1.43 ng/g fat in dairy products, closer to our values (1.4 ng/g fat) than those reported by Barone et al. [14] (13 ng/g fat), despite the proximity to an industrial area. Non-compliance rates were comparable with ours (0.6% versus our 0.46%). At the European level, Costopoulou et al. [16] reported higher contamination in Greek food samples (2002–2022), median values for fish (0.56 pg/g ww), meat (0.47 pg/g fat), milk and dairy products (0.57 pg/g fat), and eggs (0.40 pg/g fat). These differences may be attributed to the longer period, which includes earlier years with higher levels of contamination, and to the different environmental conditions in Greece. Stadion et al. [17] reported that plant-based foods show substantially lower values; the ranking among food categories, with fatty foods of animal origin, especially fish, showing the highest contamination, is consistent with the patterns observed in our dataset. Pajurek et al. [19] reported particularly low contamination in foods for infants and young children, with median PCDDs/Fs+dl-PCBs values of 0.0075–0.0197 pg WHO_05_-TEQ/g ww, confirming minimal contamination for this kind of matrix.

To facilitate systematic comparison, contamination levels are presented in Table 6.

### 4.3. Interpretation of Adult Intake of PCDD/Fs and dl-PCBs

Dietary exposure estimates were calculated to provide indicative context for interpreting the low levels of contamination observed in this study, rather than as a formal risk assessment. Given the simplified, deterministic nature of the approach and the inherent limitations of combining mean contaminant concentrations with mean consumption data, these values should be considered rough upper-limit estimates and interpreted with caution. The estimated daily intakes of 0.33 pg WHO_05_-TEQ/kg bw/day (INRAN 2005–06) and 0.36 pg/kg bw/day (CREA 2018–2020), corresponding to approximately 2.31–2.52 pg/kg bw/week, indicate that dietary exposure in Central Italy remains in the order of the EFSA TWI of 2 pg/kg bw/week. These estimates indicate that, despite the low levels of contamination documented in central Italy, dietary exposure remains in the order of the EFSA TWI rather than substantially below it. Fish and cheese were the main contributors, accounting for approximately 67–71% of the estimated total intake. Compared with previous Italian estimates, the intakes observed here are generally lower than those reported by Diletti et al. [13] for Italian adults using the same INRAN 2005–06 dataset (mean: 0.90 pg/kg body weight/day; median: 0.64 pg/kg body weight/day). Compared with Diletti et al. [13], whose data cover the period 2013–2016, the difference may reflect both a temporal decline in contamination, consistent with the progressive effects of stricter EU regulations, and the regional scope of our dataset, which is limited to central Italy.

## 5. Conclusions

Within the framework of official controls, this study offers a comprehensive ten-year (2016–2025) assessment of contamination by PCDDs/Fs, dl-PCBs, and ndl-PCBs in feed and food, highlighting significant reductions compared to previous European and Italian surveillance data.

Feed contamination was low, with median WHO_05_-TEQ values 10–100-fold lower than those reported in recent European data (2002–2010), consistent with declining trends in feed contamination over time. Food contamination was consistently 2–4 times lower than recent Italian national data (2013–2016) and lower than recent reports from Southern Italy and Greece. Species-level analysis confirmed established patterns (higher contamination in game and sheep products than in pork and poultry), but absolute levels remained very low (<0.5 pg WHO_05_-TEQ/g fw for most products). Baby foods exhibited particularly low contamination (median 0.0036 pg/g wet weight).

Temporal analysis from 2016 to 2025 revealed stable contamination, indicating that improvements achieved under EU regulations have been maintained. Current risk management measures, including maximum limits, action levels, and mandatory monitoring, continue to effectively prevent re-contamination.

Preliminary dietary exposure estimates based on measured contaminant concentrations and average daily food consumption were approximately 2.31–2.52 pg/kg bw/week. These values are slightly above the daily equivalent of the EFSA tolerable weekly intake (2 pg/kg bw/week) but substantially lower than previous national estimates. Fish and cheese remained the main contributors to exposure, reflecting the lipophilic nature of these contaminants.

In conclusion, feed and food contamination in central Italy (local and imported) is significantly lower than in previous surveillance periods, generally well below regulatory thresholds, and is associated with dietary exposure estimates that, though slightly exceeding the EFSA TWI and probably overestimated given the deterministic approach applied, remain lower than previous national estimates. These findings support the effectiveness of current EU and Italian risk management strategies and highlight the importance of maintaining surveillance. However, the variability in contamination levels observed across categories, including occasional samples with values that fall above the general distribution, while remaining compliant, underscores the need for ongoing systematic surveillance to identify emerging contamination trends.

## Figures and Tables

**Table 1:** Distribution of feed and food samples analysed between 2016 and 2025 in the Latium and Tuscany regions (Italy). The table presents the total number of samples and their distribution by region and category. Percentages for food samples are reported to two decimal places; therefore, the total exceeds 100% due to rounding. Percentages for Tuscany and Latium refer to the total number of samples within each feed or food category. The percentages for each region reflect the proportion of samples within each feed or food category.

**Feed Category**	**Frequency (N)**	**Percentage of Total (%)**	**Tuscany Frequency (N)**	**Tuscany-Percentage (%)**	**Latium** **Frequency (N)**	**Latium-** **Percentage (%)**
**Additive**	11	3	8	73	3	27
**Premix**	10	3	2	20	8	80
**Raw material**	165	42	58	35	107	65
**Compound feed**	204	52	76	37	128	63
**Total**	**390**	**100**	**144**	**37**	**246**	**63**
**Food category**	**Frequency** **(N)**	**Percentage of Total (%)**	**Tuscany** **Frequency (N)**	**Tuscany-Percentage (%)**	**Latium** **Frequency (N)**	**Latium-** **Percentage (%)**
**Meat and Meat Products**	397	22.61	205	51.64	192	48.36
**Fishery Products and Bivalve** **Molluscs**	504	28.70	137	27.18	367	72.82
**Milk and Dairy Products**	224	12.76	99	44.20	125	55.80
**Eggs**	117	6.66	46	39.32	71	60.68
**Vegetable Oils**	292	16.63	60	20.55	232	79.45
**Baby Food**	103	5.87	1	0.97	102	99.03
**Marine Oils**	48	2.73	1	2.08	47	97.92
**Animal Fats**	53	3.02	17	32.08	36	67.92
**Liver**	16	0.91	14	87.50	2	12.50
**Total**	**1756**	**100**	**580**	**33**	**1176**	**67**

**Table 2:** List of studied PCDDs, PCDFs, and dlPCBs.

Dibenzo-Para-Dioxins (PCDDs) and Dibenzo-Para-Furans (PCDFs)	Dioxin-like PCBs (dlPCBs)	Non-Dioxin-like PCBs (ndlPCBs)
2,3,7,8-TCDD	*non-ortho PCBs*	PCB 28
1,2,3,7,8-PeCDD	PCB 77	PCB 52
1,2,3,4,7,8,-HxCDD	PCB 81	PCB 101
1,2,3,6,7,8,-HxCDD	PCB 126	PCB 138
1,2,3,7,8,9-HxCDD	PCB 169	PCB 153
1,2,3,4,6,7,8-HpCDD	-	PCB 180
OCDD	*ortho PCBs*	-
2,3,7,8-TCDF	PCB 105	-
1,2,3,7,8-PeCDF	PCB 114	-
2,3,4,7,8-PeCDF	PCB 118	-
1,2,3,4,7,8-HxCDF	PCB 123	-
1,2,3,6,7,8-HxCDF	PCB 156	-
1,2,3,7,8,9-HxCDF	PCB 157	-
2,3,4,6,7,8-HxCDF	PCB 167	-
1,2,3,4,6,7,8-HpCDF	PCB 189	-
1,2,3,4,7,8,9-HpCDF	-	-
OCDF	-	-

**Table 3:** Statistical descriptors of PCDDs/Fs and dl and ndl-PCBs in various feed samples by category: WHO_05_-TEQ values of PCDDs/Fs and dl-PCBs and their sum are in ng WHO_05_-TEQ/kg feed, normalised to 12% moisture; ndl-PCBs values are in μg/Kg feed, normalised to 12% moisture.

Feed Category	Sum	Mean	Median	P25	P75
**Additive**	PCDDs/Fs+dl-PCBs	0.020	0.0090	0.0060	0.014
	PCDDs/Fs	0.015	0.0070	0.0040	0.011
	dl-PCBs	0.019	0.0020	0.0010	0.0050
	ndl-PCBs	0.56	0.060	0.030	0.21
**Premix**	PCDDs/Fs+dl-PCBs	0.030	0.014	0.0070	0.030
	PCDDs/Fs	0.020	0.0080	0.0060	0.016
	dl-PCBs	0.0050	0.0020	0.0010	0.0050
	ndl-PCBs	0.19	0.11	0.050	0.15
**Raw material**	PCDDs/Fs+dl-PCBs	0.13	0.020	0.010	0.080
	PCDDs/Fs	0.060	0.010	0.0060	0.030
	dl-PCBs	0.070	0.0080	0.0030	0.040
	ndl-PCBs	1.0	0.10	0.050	0.24
**Compound feed**	PCDDs/Fs+dl-PCBs	0.11	0.017	0.0082	0.17
	PCDDs/Fs	0.040	0.0090	0.0050	0.030
	dl-PCBs	0.080	0.0070	0.020	0.10
	ndl-PCBs	0.87	0.13	0.050	0.95

**Table 4:** Statistical descriptors of PCDD/Fs, dl- and ndl-PCBs in various food samples by category. PCDD/Fs and dl-PCBs: pg WHO_05_-TEQ/g fw; ndl-PCBs: ng/g fw. Exceptions for fish, seafood, liver and baby food: values on wet weight (ww).

Food Category	Sum	Mean	Median	P25	P75
**Meat And Meat Products**	PCDDs/Fs+dl-PCBs	0.19	0.090	0.045	0.24
	PCDDs/Fs	0.089	0.052	0.029	0.098
	dl-PCBs	0.096	0.023	0.0090	0.090
	ndl-PCBs	1.3	0.66	0.26	1.5
**Fishery Products and Bivalve Molluscs**	PCDDs/Fs+dl-PCBs	0.16	0.096	0.038	0.18
	PCDDs/Fs	0.032	0.018	0.010	0.030
	dl-PCBs	0.13	0.071	0.018	0.15
	ndl-PCBs	1.6	0.77	0.29	1.5
**Milk And Dairy Products**	PCDDs/Fs+dl-PCBs	0.42	0.32	0.11	0.55
	PCDDs/Fs	0.11	0.073	0.041	0.14
	dl-PCBs	0.30	0.20	0.040	0.46
	ndl-PCBs	3.7	1.4	0.67	3.0
**Eggs**	PCDDs/Fs+dl-PCBs	0.19	0.12	0.083	0.18
	PCDDs/Fs	0.11	0.073	0.050	0.11
	dl-PCBs	0.076	0.034	0.016	0.067
	ndl-PCBs	1.4	1.0	0.56	1.9
**Vegetable Oils**	PCDDs/Fs+dl-PCBs	0.061	0.044	0.031	0.063
	PCDDs/Fs	0.041	0.031	0.023	0.043
	dl-PCBs	0.023	0.011	0.0059	0.018
	ndl-PCBs	0.21	0.090	0.050	0.16
**Baby Food**	PCDDs/Fs+dl-PCBs	0.0067	0.0036	0.0019	0.0064
	PCDDs/Fs	0.0044	0.0024	0.0014	0.0045
	dl-PCBs	0.0025	0.00090	0.00030	0.0022
	ndl-PCBs	0.059	0.020	0.010	0.050
**Marine Oils**	PCDDs/Fs+dl-PCBs	0.36	0.15	0.070	0.54
	PCDDs/Fs	0.072	0.047	0.026	0.093
	dl-PCBs	0.29	0.081	0.028	0.48
	ndl-PCBs	4.2	2.7	1.1	5.7
**Animal Fats**	PCDDs/Fs+dl-PCBs	0.088	0.055	0.035	0.091
	PCDDs/Fs	0.056	0.035	0.025	0.059
	dl-PCBs	0.032	0.013	0.0064	0.024
	ndl-PCBs	1.0	0.32	0.22	0.43
**Liver**	PCDDs/Fs+dl-PCBs	0.036	0.049	0.023	0.24
	PCDDs/Fs	0.021	0.013	0.010	0.021
	dl-PCBs	0.15	0.027	0.0059	0.083
	ndl-PCBs	0.20	0.12	0.078	0.15

**Table 5:** Dietary intake estimates based on the INRAN-SCAI [29] and CREA [30] food consumption surveys.

Categories	INRAN 2005–06	Contribution INRAN (%)	CREA 2018–2020	Contribution CREA (%)
Eggs	0.00696	2	0.00766	2
Fish & seafood	0.153	46	0.194	54
Meat	0.0125	4	0.015	4
Milk, whey & cream	0.0347	11	0.0348	10
Butter	0.0208	6	0.0175	5
Cheese	0.0702	21	0.0629	17
Vegetable fats & oils	0.0319	10	0.0259	7
**Total**	**0.33**	**100**	**0.36**	**100**

**Table 6:** Comparison of median values of PCDDs/Fs and PCBs in food with Italian and Mediterranean studies. PCDDs/Fs and dl-PCBs: pg WHO_05_-TEQ/g fw; ndl-PCBs: ng/g fw. Exceptions for fish, seafood, liver, and baby food: values on wet weight (ww). Where only mean values were available, this is indicated as (mean).

Study	Meat	Fish	Milk & Dairy	Eggs	Veg. Oils	Liver	Baby Food
***PCDD/Fs+dl-PCBs***							
This study (2016–2025)	0.090	0.096	0.32	0.12	0.044	0.049	0.0036
Diletti et al. [13] (2013–2016)	0.41	0.31	0.64	0.36	0.081 ^a^	0.88 ^b^ (mean)	—
Barone et al. [14] (May–July 2019)	1.70	0.50	1.37–2.20 ^a^	0.71	0.09–0.33 ^a^	—	—
Barone et al. [20] (March–April 2018)	1.8 (mean)	—	—	—	—	—	—
Giannico et al. [18] (2013–2018)	—	—	0.73 ^a^	—	—	—	—
Costopoulou et al. [16] (2002–2022)	0.47 ^a^	0.56	0.57 ^a^	0.40	0.34 ^a^	—	—
Stadion et al. [17] (2022)	0.258	0.903	0.523	0.187	0.430	—	—
Pajurek et al. [19] (2023)	—	—	—	—	—	—	0.0075–0.0197
***ndl-PCBs***							
This study (2016–2025)	0.66	0.77	1.4	1.0	0.09	—	—
Barone et al. [15] (May–July 2019)	30 (mean)	3.5 (mean)	13 (mean)	10 (mean)	2.8 (mean)	—	—
Barone et al. [20] (March–April 2018)	45 (mean)	—	—	—	—	—	—
Costopoulou et al. [16] (2002–2022)	2.8 (mean)	5.3 (mean)	2.4 ^a^ (mean)	1.6 (mean)	2.5 (mean)	—	—
Giannico et al. [18] (2013–2018)	—	—	1.68 ^a^	—	—	—	
Pajurek et al. [19] (2023)	—	—	—	—	—	—	0.021–0.057

Notes: ^a^ Values represent medians calculated from the collected data. When categories were reported separately, the final value corresponds to the average of the category medians. ^b^ sheep liver only.

## Data Availability

The original contributions presented in the study are included in the article, further inquiries can be directed to the corresponding author.

## Abbreviations

The following abbreviations are used in this manuscript:

DSCF	Dwass–Steel–Critchlow–Fligner test
PCDDs/Fs	Polychlorinated Dibenzo-p-Dioxins and Furans
PCBs	Polychlorinated Biphenyls
WHO_05_-TEQ	World Health Organisation Toxic Equivalence
TEF	Toxic Equivalency Factors
ww	Wet weight
fw	Fat weight

## Appendix A

**Table A1:** Kruskal–Wallis test results for feed raw materials by origin. Contamination was compared across plant (n = 135), animal (n = 29), and mineral (n = 2) raw materials using the Kruskal–Wallis test. No significant differences were observed for any contaminant class.

Values	χ^2^	df	*p *	Interpretation
ng WHO-PCDDs/Fs+dl-PCBs-TEQ/kg	0.859	2	0.651	Non-significant
ng WHO-PCDDs/Fs-TEQ/kg	2.048	2	0.359	Non-significant
ng WHO-PCBs-TEQ/kg	0.177	2	0.915	Non-significant
Ndl-PCBs (µg/Kg)	1.894	2	0.388	Non-significant

χ^2^ = Kruskal–Wallis test statistic; df = degrees of freedom. Statistical significance set at *p* < 0.05.

**Table A2:** Kruskal–Wallis test results for (a) complete vs. compound feed (b) Dwass–Steel–Critchlow–Fligner (DSCF) test statistic for complete feed across different specie.

(a) Kruskal–Wallis test results for feed. Contamination was compared across complete feeds (n = 129) and complementary feeds (n = 74) using the Kruskal–Wallis test.				
**Values**	** χ^2^**	**df**	*** p ***	** Interpretation **
ng WHO-PCDDs/Fs+dl-PCBs-TEQ/kg	20.8	1	<0.001	Significant
ng WHO-PCDDs/Fs-TEQ/kg	15.4	1	<0.001	Significant
ng WHO-PCBs-TEQ/kg	25.5	1	<0.001	Significant
Ndl-PCBs (µg/Kg)	25.0	1	<0.001	Significant
(b) DSCF test statistic for WHO-PCDD/F+dl-PCB-TEQ (ng/kg, 12% moisture): for complete feeds by species destination. Sample sizes: Aquaculture (n = 56), Bovines (n = 24), Poultry (n = 27)				
		** W **	*** p ***	** Interpretation **
Bovines	Aquaculture	9.6958	<0.001	Significant
Bovines	Poultry	−1.1324	0.997	Non-significant
Aquaculture	Poultry	−8.0269	<0.001	Significant

Notes: Samples with low representativity were not included in the DSCF test. Statistical significance set at *p* < 0.05.

**Table A3:** DSCF pairwise comparison test for meat types. Parameter: WHO_05_-PCDD/F+dl-PCB-TEQ (pg/g fw). Sample sizes: Ovine (n = 51), Bovine (n = 82), Pig (n = 146), Poultry (n = 107).

		W	*p*	Interpretation
Pig	Bovine	5.551	0.001	Significant
Pig	Ovine	7.087	<0.001	Significant
Pig	Poultry	−0.130	1.000	Non-significant
Bovine	Ovine	0.628	0.998	Non-significant
Bovine	Poultry	−5.997	<0.001	Significant
Ovine	Poultry	−6.737	<0.001	Significant

**Table A4:** DSCF pairwise comparison test for milk and dairy products. Parameter: WHO_05_-PCDD/F+dl-PCB-TEQ (pg/g fw). Sample sizes: Ovine (n = 82), Cow/buffalo (n = 79), Cheese (n = 27), Butter (n = 13).

		W	*p*	Interpretation
Butter	Cheese	1.470	0.726	Non-significant
Butter	Cow/Buffalo	1.704	0.624	Non-significant
Butter	Ovine	3.469	0.068	Non-significant
Cheese	Cow/Buffalo	0.446	0.989	Non-significant
Cheese	Ovine	3.281	0.094	Non-significant
Cow/Buffalo	Ovine	3.369	0.081	Non-significant

**Table A5:** DSCF pairwise comparison test for fish and molluscs. Parameter: WHO_05_-PCDD/F+dl-PCB-TEQ (pg/g ww). Sample sizes: Unclassified * (n = 25), Molluscs (n = 299), Medium-fat fish (n = 85), Fatty fish ^‡^ (n = 14), Lean fish (n = 65), Crustaceans (n = 16). * Unclassified category consists primarily of heterogeneous samples from the 2016–2017 sampling period. ^‡^ Fatty fish category dominated by farmed salmon (11/14 samples).

		W	*p*	Interpretation
Crustaceans	Molluscs	5.0961	0.004	Significant
Crustaceans	Fatty fish	3.3510	0.167	Non-significant
Crustaceans	Lean fish	0.4865	0.999	Non-significant
Crustaceans	Unclassified fish	6.9171	<0.001	Significant
Crustaceans	Medium-fat fish	6.3138	<0.001	Significant
Molluscs	Fatty fish	0.0684	1.000	Non-significant
Molluscs	Lean fish	−7.1221	<0.001	Significant
Molluscs	Unclassified fish	7.5152	<0.001	Significant
Molluscs	Medium-fat fish	6.1119	<0.001	Significant
Fatty fish	Lean fish	−3.1504	0.225	Non-significant
Fatty fish	Unclassified fish	3.7060	0.092	Non-significant
Fatty fish	Medium-fat fish	2.0024	0.718	Non-significant
Lean fish	Unclassified fish	7.1791	<0.001	Significant
Lean fish	Medium-fat fish	7.8497	<0.001	Significant
Unclassified fish	Medium-fat fish	−3.8937	0.065	Non-significant
